# Benefit of a flash dose of corticosteroids in digestive surgical oncology: a multicenter, randomized, double blind, placebo-controlled trial (CORTIFRENCH)

**DOI:** 10.1186/s12885-022-09998-z

**Published:** 2022-08-23

**Authors:** Joséphine Magnin, Isabelle Fournel, Alexandre Doussot, Jean-Marc Régimbeau, Philippe Zerbib, Guillaume Piessen, Laura Beyer-Berjot, Sophie Deguelte, Zaher Lakkis, Lilian Schwarz, David Orry, Ahmet Ayav, Fabrice Muscari, François Mauvais, Guillaume Passot, Nelson Trelles, Aurélien Venara, Stéphane Benoist, Mathieu Messager, David Fuks, Baptiste Borraccino, Christophe Trésallet, Alain Valverde, François-Régis Souche, Astrid Herrero, Sébastien Gaujoux, Jérémie Lefevre, Abderrahmane Bourredjem, Amélie Cransac, Pablo Ortega-Deballon

**Affiliations:** 1 Service de Chirurgie Digestive et Cancérologique, CHU François Mitterrand, 14 rue Paul Gaffarel, 21000 Dijon, France; 2 Department of Digestive Surgical Oncology, University Hospital of Dijon, INSERM 1432, University of Bourgogne, Dijon, France; 3 Department of Clinical Epidemiology, University Hospital of Dijon, INSERM CIC 1432, University of Bourgogne, Dijon, France; 4 Department of Digestive Surgical Oncology and Liver Transplantation, University Hospital of Besançon, Besançon, France; 5 Department of Digestive Surgical Oncology, University Hospital of Amiens, Amiens, France; 6 Department of Digestive Surgical Oncology and Liver Transplantation, Claude Huriez University Hospital, Chu Lille, France; 7 Department of Digestive and Oncological Surgery, Claude Huriez University Hospital, Chu Lille, France; 8 Department of Digestive Surgical Oncology, North University Hospital, Marseille, France; 9 Department of Digestive Surgical Oncology, University Hospital of Reims, Reims, France; 10 Department of Digestive Surgical Oncology, University Hospital of Rouen, Rouen, France; 11 Department of Surgical Oncology, Georges François Leclerc Cancer Center, Dijon, France; 12 Department of Digestive Surgical Oncology, University Hospital of Nancy, Nancy, France; 13 Department of Digestive Surgical Oncology, Rangueil University Hospital, Toulouse, France; 14 Department of Digestive Surgery, Simone Veil Hospital, Beauvais, France; 15 Department of Digestive Surgical Oncology, Pierre Bénite University Hospital, Lyon, France; 16 Department of Digestive Surgery, René-Dubos Hospital, Cergy-Pontoise, France; 17 Department of Digestive Surgical Oncology, University Hospital of Angers, Angers, France; 18 Department of Digestive Surgical Oncology, Bicêtre University Hospital, Le Kremlin-Bicêtre, France; 19 Department of Digestive Surgery, Gustave Dron Hospital, Tourcoing, France; 20 Department of Digestive Surgical Oncology, Cochin University Hospital, Paris, France; 21 Department of Digestive Surgery, Hospital of Auxerre, Auxerre, France; 22 Department of Digestive Surgical Oncology, Avicenne University Hospital, Paris, France; 23 Department of Digestive Surgery, La Croix Saint Simon Hospital, Paris, France; 24 Department of Digestive Surgical Oncology, University Hospital of Montpellier, Montpellier, France; 25 Department of Digestive Surgical Oncology and Liver Transplantation, University Hospital of Montpellier, Montpellier, France; 26 Department of Digestive Surgical Oncology, Pitié Salpêtrière University Hospital, Paris, France; 27 Department of Digestive Surgical Oncology, Saint-Antoine University Hospital, Paris, France; 28 Department of Pharmacy, University Hospital of Dijon, Dijon, France

**Keywords:** Perioperative corticosteroids, Digestive surgical oncology, Randomized placebo-controlled trial, Postoperative morbidity, cancer-related outcomes

## Abstract

**Background:**

The modulation of perioperative inflammation seems crucial to improve postoperative morbidity and cancer-related outcomes in patients undergoing oncological surgery. Data from the literature suggest that perioperative corticosteroids decrease inflammatory markers and might be associated with fewer complications in esophageal, liver, pancreatic and colorectal surgery. Their benefit on cancer-related outcomes has not been assessed.

**Methods:**

The CORTIFRENCH trial is a phase III multicenter randomized double-blind placebo-controlled trial to assess the impact of a flash dose of preoperative corticosteroids versus placebo on postoperative morbidity and cancer-related outcomes after elective curative-intent surgery for digestive cancer. The primary endpoint is the frequency of patients with postoperative major complications occurring within 30 days after surgery (defined as all complications with Clavien-Dindo grade > 2). The secondary endpoints are the overall survival at 3 years, the disease-free survival at 3 years, the frequency of patients with intraabdominal infections and postoperative infections within 30 days after surgery and the hospital length of stay. We hypothesize a reduced risk of major complications and a better disease-survival at 3 years in the experimental group. Allowing for 5% of drop-out, 1 200 patients (600 per arm) should be included.

**Discussion:**

This will be the first trial focusing on the impact of perioperative corticosteroids on cancer related outcomes. If significant, it might be a strong improvement on oncological outcomes for patients undergoing surgery for digestive cancers.

**Trial registration:**

ClinicalTrials.gov, NCT03875690, Registered on March 15, 2019, URL: https://clinicaltrials.gov/ct2/show/NCT03875690.

**Supplementary Information:**

The online version contains supplementary material available at 10.1186/s12885-022-09998-z.

## Background

Inflammation is harmful in cancer patients, especially in those undergoing surgery. It increases the risk of postoperative complications, time to recovery, hospital length of stay and impairs cancer-related outcomes (recurrence and survival) [1–7]. High preoperative levels of C-reactive protein and pro inflammatory adipocytokines are associated with a higher risk of postoperative morbidity in colorectal cancer surgery [8–10]. Perioperative inflammation is also a predictor of poorer survival in patients operated for digestive cancer whether complications occur or not [11–19]. So, the modulation of surgery-induced inflammation seems crucial to improve cancer survival.

The effects of a preoperative high dose of corticosteroids (flash) over the inflammatory markers have been established in different surgical fields [20–25], as well as its safety, namely in digestive surgery regarding the onset of anastomotic leakage and surgical site infection [20, 22–24]. Two systemic reviews and meta-analyses showed that perioperative corticosteroids decreased inflammatory markers and were associated with a diminution until 50% of the risk of some postoperative complications after esophageal, liver, pancreatic and colorectal surgery [24, 25]. Regarding the dose used, a few studies have used dexamethasone or hydrocortisone at low doses [26, 27], but most of the trials have used methylprednisolone at high doses, ranging between 500 mg independently of the patient’s weight to 30 mg/kg [24, 25, 28–34].

In most studies, the main endpoint was the concentration of inflammatory markers [24, 25] with little attention paid to clinically pertinent criteria or the prognosis of cancer, although the literature suggests that reducing the incidence of severe complications will improve the oncological outcomes [7, 8, 29, 30].

Thus, a prospective randomized controlled trial is warranted in order to evaluate the safety and the benefit of a flash dose of corticosteroids on postoperative morbidity and cancer-related outcomes after surgery for digestive cancer.

## Methods/design

### Protocol overview

**Fig. 1:**
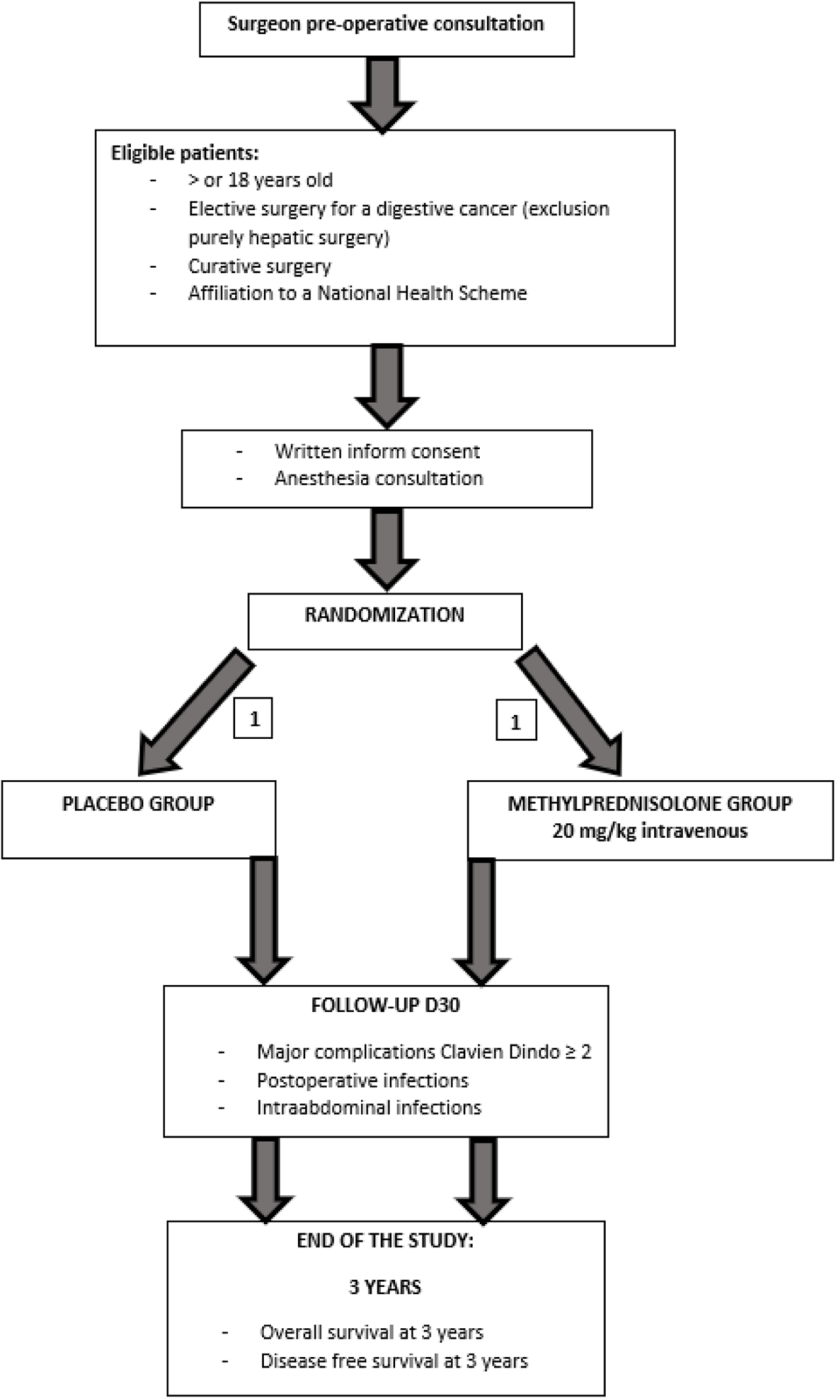
Flowchart of the trial

The CORTIFRENCH trial is a phase III, multi center, randomized, double-blind, placebo-controlled trial to assess the superiority of a flash dose of preoperative corticosteroids on the reduction of major complications after digestive surgical oncology. Patients likely to be included in this trial will be randomized in a 1:1 ratio between two groups: an experimental group receiving 20 mg/kg of methylprednisolone intravenous at the time of anesthesia induction and a control group receiving an intravenous placebo with an identical aspect. All members of the anesthesiology and surgical team will be unaware of the group assignments, as well as the investigator and the medical team managing the patient and recording the outcomes. Major complications (Clavien-Dindo grade > 2) [35] occurring within 30 days after surgery, postoperative and intra-abdominal infections at postoperative day 30 (D30) will be recorded and overall and disease-free survival at 3 years will be evaluated in order to assess if a flash dose of preoperative corticosteroids has a real impact on the onset of postoperative complications, recovery and cancer-related outcomes after digestive cancer surgery. The flowchart of the study has been reported in Fig. 1.

### Objectives

The primary objective is to assess the impact of a flash dose of preoperative corticosteroids versus placebo on the onset of major complications (Clavien-Dindo > 2) [35] within 30 days after elective curative-intent surgery for digestive cancer.

Secondary objectives are to assess the safety of a preoperative flash of corticosteroids versus placebo and its influence on postoperative and intra-abdominal infections at D30, hospital length of stay and 3-year overall and disease-free survival.

### Inclusion criteria

Inclusion criteria are patients older than 18 years old, undergoing elective surgery for a digestive cancer (except purely hepatic surgery), operated in a curative intent, affiliated to the French National Health System and providing their written informed consent.

### Exclusion criteria

Patients with any of the following criteria will not be included in the study: emergency surgery, palliative surgery, exclusive liver surgery, surgery with concomitant hyperthermic intraperitoneal chemotherapy, pregnant or breastfeeding women, ASA score > 3, long-term ongoing oral treatments with steroids, a contra-indication to steroids therapy (active infection, ongoing viral disease, uncontrolled psychotic state, hyper sensitivity to methylprednisolone or to one of his excipients), impossibility to adhere to the medical follow-up of the trial for geographical, social or psychological reasons, patients subject to a measure of legal protection (guardianship, tutorship) and persons subject to a court order. Patients with live vaccine or live attenuated vaccine administered within 1 month before surgery and cancelled surgery without deferral will be secondary excluded.

### Endpoints

The primary endpoint is the frequency of patients with postoperative major complications occurring within 30 days after surgery (D30). Major complications are defined as all complications with Clavien-Dindo grade > 2 [35].

Secondary endpoints are:

- the overall survival at 3 years (defined as the time from surgery to death from any cause),
- the disease-free survival at 3 years (defined as the time from surgery to first documented progressive disease or death from any cause, whichever occurs first),
- the frequency of patients with postoperative infections occurring within 30 days after surgery and defined according to the CDC (Centers for Disease Control and Prevention) definitions,
- the frequency of patients with intra-abdominal infections (including anastomotic fistula and intraabdominal abscess) within 30 days after surgery and defined according to the CDC definitions,
- the hospital length of stay (in the case of death, the patient will be considered as hospitalized until D30),
- the frequency and the type of side effects, particularly hyperglycemia, electrolyte disorders and wound healing assessed respectively by glycemia and electrolyte panel within the first 24 postoperative hours and clinical inspection at D30 follow-up visit.

### Randomization

Eligible patients will be identified at the preoperative consultation with the surgeon. If the patient meets the eligible criteria, the investigating doctor will present the study. The patient will have a reflection period after the consultation and will be informed of his/her right to withdraw consent at any time without prejudice and without having to justify this decision. After receiving the written informed consent and after verification of the inclusion criteria, the patient will be included.

Patients will be randomized in a 1:1 ratio between 2 groups receiving either 20 mg/kg of intravenous methylprednisolone (experimental group) or an identical-aspect intravenous placebo (control group) at the time of anesthesia induction.

Randomization will be performed online by the investigator using the secure CleanWeb platform at the time of inclusion and stratified on the center, the site of surgery (upper digestive/pancreatic or colorectal cancer) and the surgical approach (scheduled laparoscopic or open surgery).

### Blinding process

To maintain a double-bind trial, all members of the anesthesiology and surgical team will be unaware of the group assignments. Only the pharmacy of each participating center will know the randomization group in order to prepare the bag to be infused and deliver it to the anesthesiology team. If the pharmacy is not able to prepare the allocated treatment for logistical reasons, a member of the nursing staff will be authorized to carry out the preparation. Then, this person will not be involved in the follow-up of the patient to maintain the double blind.

Blind may be lifted in case of unexplained or possible toxic death, in case of a serious adverse event when the knowledge of the product administered is necessary for the care of the patient and if the medical care is different depending on the treatment receive, in the event of accidental or intentional taking by a person other than the participant tested and in the case of a suspected unexpected serious adverse reaction for the purpose of transmission to authorities.

### Treatment methods

Patients randomized in the experimental group will receive 20 mg/kg intravenous methylprednisolone infused in a ready-to-use 50 mL bag of sodium chloride 0.9% during 30 min at the time of anesthetic induction.

Patients randomized in control/placebo group will receive 50 mL of intravenous sodium chloride 0.9% in a ready-to-use bag during 30 min at the time of anesthetic induction. As methylprednisolone is colorless, bags containing sodium chloride alone or sodium chloride plus methylprednisolone will identical aspect.

Live vaccines or live attenuated vaccines will be contra-indicated during the month preceding surgery and until 3 months later.

### Surgical management

#### Pre-operative check-up

Each institution will prepare patients and scheduled surgery according to its own institutionally validated protocols. All patients included will undergo the preoperative evaluation currently performed at each institution. Preoperative immunonutrition will not be mandatory but its use will be recorded.

#### Intraoperative management

At the time of the anesthesia induction, methylprednisolone or placebo will be administrated according to the randomization group. Diabetics patients will be closely monitored in both groups during and immediately after surgery by capillary glycemia every 3 h in order to adjust insulin therapy as appropriate. Patients considered at risk for postoperative nausea and emesis will receive a single preoperative dose of 4–8 mg of dexamethasone intravenous (equivalent to 20–40 mg of methylprednisolone), following the guidelines of the French Society of Anesthesiology and Reanimation (SFAR). This information will be recorded.

#### Postoperative management

Clinical and biological follow-up (blood samples) will be performed according to the protocols of each institution. The only specific requirement of the study will be an electrolyte panel and fasting glycemia performed within the first 24 h after surgery in order to detect a possible effect of corticosteroids. The postoperative management, monitoring and follow-up will be as usual according to each institution’s current practices, but each participating center will be asked to record the following data: day of oral refeeding, mean daily pain visual analog scale, onset of fever or any complication (whether infectious or not), laboratory and imaging studies performed between surgery and postoperative day 30.

### Follow-up

According to each center’s protocol, a D30 visit will be scheduled between postoperative days 28 and 45. Patients will undergo a clinical evaluation by the surgeon and will be asked about any events occurring since the initial hospital discharge. Any complications occurred since the initial discharge or detected during the examination will be recorded. In case of re-hospitalization between hospital discharge and the scheduled follow-up visit at postoperative D30, all data related to any complications will be collected. Dead patients will be considered to have been hospitalized between the date of death and postoperative D30.

The study will end at 3 years. At the end of the study, vital status (including date and cause of death if appropriate) and information about disease progression (including the date of discovery in case of recurrence) will be collected and recorded for all included patients.

### Data collection

The data will be entered into an e-CRF (electronic Case Report Form) specifically developed for this study using a Clinical Data Management System (CMDS-CleanWeb). All required information for the study will be entered as and when it is obtained. Thanks to automatic checks, in case of missing or inconsistent data, request for correction will be sent to participating centers via the CMDS. If corrections are necessary, they will be made directly using the CMDS. At the end of the study, a blind review of data will be performed and in case of additional queries, it should be resolved before the final database lock.

### Statistical analysis and sample size

This study is a phase III multi center randomized double blind placebo-controlled trial. According to the literature, the incidence of major complications at D30 after surgery is 26% [36]. We hypothesized this proportion will decrease to 18% in patients with corticosteroids administration. Based on these hypotheses with an alpha risk of 5% and a power of 90% and including an interim analysis at the half of the inclusions, 1184 analyzable patients (592 per arm) are required. Allowing for 5% of drop-out, 1200 patients (600 per arm) will be included. Statistical analyses will be performed at the coordinating center (INSERM CIC 1432 of Dijon).

#### Demographics and baseline characteristics

Patient’s characteristics at baseline will be described in terms of frequencies for categorical variables and in terms of means (+/- standard deviation) or medians (interquartile range) for continuous variables. These characteristics will be compared between groups using Chi squared (or Fisher’s exact test) or t-Test (or Mann-Whitney test) when appropriate.

#### Interim analysis

One interim analysis will be performed after the inclusion of 592 patients (296 per arm). The interim analysis will assess the superiority of the experimental arm versus the control group for the primary endpoint: early termination will be considered only if superiority is met. The interim analysis will have a two-sided alpha level of 0.0003. According to the O’Brien-Fleming spending rule [37], this will leave a two-sided alpha level of 0.049 for the final analysis. The data safety monitoring board will decide if the trial should be stopped according to the results of the interim analysis.

#### Main analysis

The main analysis will be conducted on intention-to-treat (ITT) analysis. The proportion of patients with postoperative major complications at D30 will be compared using Chi-square test. If there are any differences between the two groups concerning the baseline characteristics, a logistic regression analysis including confounding factors will allow us to check the robustness of the conclusion based on the main analysis. Patients with missing data on adjustment variables will be excluded from multivariate analysis. Results will be expressed using Odds ratios and their 95% confidence intervals. Patients lost to follow-up will be first analyzed successively under the maximal bias analysis (postoperative major complications in corticosteroids patients and no major complications in placebo patients) and then excluded. This ITT analysis will be completed with an m-ITT analysis (exclusion of all subjects in the ITT population who don’t meet inclusion and non-inclusion criteria) and with a per-protocol (PP) analysis (exclusion of all subjects in the ITT population who meet any of the following criteria: no study treatment administered or major protocol deviation).

#### Secondary analysis

The frequency of patients with postoperative infections and intra-abdominal infections will be compared between groups according to the same strategy as the main analysis (bivariate comparisons using Chi-square, multivariate analysis using logistic regression). The number of days without hospitalization at D30 will be expressed as means +/- SD or as medians (IQR) as appropriate. The Kaplan Meir methods will be used to estimate the overall and the disease-free survival curves. The corresponding survival rates will be calculated at 3 years. Median survival will also be calculated and differences between survival curves assessed using log rank test. A Cox regression analysis will be used to assess the impact of potential prognostic factors on overall survival and disease-free survival at 3 years. Subgroup analyses according to the stratification groups (site of surgery, surgical approach) will be performed for exploratory purposes. Descriptive statistics will be used to summarize adverse events and compared between the two groups. Safety analyses will be performed on the safety population which will comprise all randomized patients who consented to participate in the study and who have been given a treatment.

### Participating centers

Twenty-three French centers will participate in this study: University Hospital of Amiens, University Hospital of Dijon, University Hospital of Besançon, Georges François Leclerc Cancer Center in Dijon, Hospital of Auxerre, Simone Veil Hospital in Beauvais, Bicêtre University Hospital in Le Kremlin-Bicêtre, Claude Huriez University Hospital in Lille, La Croix Saint Simon Hospital in Paris, University Hospital of Reims, Gustave Dron Hospital in Tourcoing, Pierre Benite University Hospital in Lyon, North University Hospital in Marseille, Cochin University Hospital in Paris, René-Dubos Hospital in Cergy-Pontoise, University Hospital of Nancy, Saint-Antoine University Hospital in Paris, Avicenne University Hospital in Paris, Rangueil University Hospital in Toulouse, University Hospital of Angers, University Hospital of Montpellier, Pitié Salpêtrière University Hospital in Paris, University Hospital of Rouen.

### Ethical and safety approvals

This study protocol was approved on March 8th, 2019 by the Institutional Review Board of the Auvergne-Rhone-Alpes ethic committee. The institutional promoter is the University Hospital of Dijon. The Agence Nationale de Securité des Médicaments et des Produits de Santé gave its authorization for the study on March 12th, 2019. The study will be conducted in accordance with the ethical principles of the Declaration of Helsinki and the recommendations of the Good Clinical Practices guidelines.

## Discussion

Current evidence suggests a strong impact of perioperative inflammation on postoperative outcomes, including morbidity and cancer-related survival [1–7, 28, 29]. According to previous studies showing that a lower inflammatory activity leads to improved postoperative morbidity, different approaches have been raised to modulate perioperative inflammation, namely the use of specific anesthetics [38], preoperative immunonutrition [39, 40], or perioperative corticosteroids [8, 10, 23, 24]. Preliminary results suggest lower postoperative morbidity and an improved recovery. A flash dose of corticosteroids administered at the time of surgery could improve lung function and decrease pain, fatigue, anorexia and ileus, leading to enhanced postoperative recovery and decreased hospital stay. Patients could also begin adjuvant treatment earlier after surgery and overall and disease-free survival might be improved.

Several highlights should be emphasized regarding this trial. First, its design (randomized, placebo-control, double-blind, multicentric) will provide high-quality evidence in this field. Moreover, all endpoints will be clinically significant rather than intermediate biomarkers; some of them will focus on postoperative complications and others on cancer prognosis. Twenty-three French medical institutions will include patients in a pragmatic way: each institution will manage his/her patient according to its own protocols, except for the administration of the methylprednisolone or the placebo and the necessary surveillance of glycemia and electrolytes within the first 24 postoperative hours. To avoid excessive heterogeneity within the surgical population, we focus only on digestive cancer surgery [41]. Registering preoperative inflammatory markers will also permit to detect a potential influence on postoperative outcomes.

We will voluntarily exclude purely hepatic surgery because in such a setting, the benefit of the preoperative flash dose of corticosteroids might be specifically related to the hepatic ischemia/reperfusion induced by clamping and measured in terms of liver function [42, 43]. The specific of hepatic tolerance to clamping is beyond the scope of this trial whose main aim is to assess the flash dose of corticosteroids as a new standard in perioperative medicine.

Regarding the dose of corticosteroids we chose, Mc Sorley et al. stated in their review safety and efficacy of methyldprenisolone with doses ranging between 10 and 30 mg/kg [23]. We fond 20 mg/kg of methylprednisolone is a good compromise.

Regarding the potentials adverse outcomes induced by a flash dose of corticosteroids, the benefit/risk ratio seems to be strongly in favor of a potential benefit [24, 44, 45]. Indeed, a single high dose of corticosteroids should have no impact on the healing process or the risk of infection because these side effects do exist only with long-course therapy. The most frequent expected adverse effects are hyperglycemia and hypokalemia. To prevent these complications glycemia and kaliemia will be monitored immediately after the operation and the following day.

To conclude, in the CORTIFRENCH randomized double-blind placebo-controlled trial, we aim to show that a flash dose of corticosteroids at the time of surgery is safe, improves the postoperative recovery and the cancer-related outcomes in patients operated for a digestive cancer.

### Trial status

The trial is registered at ClinicalTrials.gov with the identifier NCT03875690. The trial is currently ongoing. The recruitment of subjects began in July 2019 and is expected to finish in September 2026.

## Supplementary Information

**Additional file 1.** CORTIFRENCH Trial: List of investigators.

## Data Availability

Indirect nominative data can’t be shared publicly under French laws. The study team is available to collaboration with other research teams on reasonable request to access study data. Expressions of interest to access study data, made out to the corresponding author, will be considered and then group-level or individual-level deidentified data may be shared as appropriate.

## Abbreviations

ASA: American Society of Anesthesiologists
CDC: Center for Disease Control and Prevention
eCRF: Electronic Case Report Form
CMDS: Clinical Data Management System
D30: postoperative day 30
IQR: Interquartile Range
SD: Standard Deviation
