# Supplementary material for: Benefit of a flash dose of corticosteroids in digestive surgical oncology: a multicenter, randomized, double blind, placebo-controlled trial (CORTIFRENCH)
Source: BMC Cancer. 2022 Aug 23;22:913. doi: 10.1186/s12885-022-09998-z (PMC9400297; doi:10.1186/s12885-022-09998-z)
Supplement: Supplementary file 1 — Additional file 1. CORTIFRENCH Trial: List of investigators. [file 12885_2022_9998_MOESM1_ESM.docx]

| **Last Name**  **CORTIFRENCH Trial: List of investigators** | **First Name** | **Academic Degree** | **Institution** | **Department** |
| --- | --- | --- | --- | --- |
| Ortega Deballon | Pablo | MD, PhD | University Hospital of Dijon, France | Digestive Surgical Oncology |
| Bouhemad | Belaïd | MD, PhD | University Hospital of Dijon, France | Anesthesiology |
| Régimbeau | Jean-Marc | MD, PhD | University Hospital of Amiens, France | Digestive Surgical Oncology |
| Badaoui | Rachid | MD | University Hospital of Amiens, France | Anesthesiology |
| Zerbib | Philippe | MD, PhD | Claude Huriez University Hospital, Lille, France | Digestive Surgical oncology and Liver transplantation |
| Lebuffe | Gilles | MD, PhD | Claude Huriez University Hospital, Lille, France | Anesthesiology |
| Piessen | Guillaume | MD, PhD | Claude Huriez University Hospital, Lille, France | Digestive and oncological surgery |
| Beyer Berjot | Laura | MD, PhD | North University Hospital, Marseille, France | Digestive Surgical Oncology |
| Pastene | Bruno | MD | North University Hospital, Marseille, France | Anesthesiology |
| Deguelte | Sophie | MD | University Hospital of Reims, France | Digestive Surgical Oncology |
| Bankole | Ezechiel | MD | University Hospital of Reims, France | Anesthesiology |
| Doussot | Alexandre | MD, PhD | University Hospital of Besançon, France | Digestive Surgical Oncology and Liver Transplantation |
| Merle | Emilie | MD | University Hospital of Besançon, France | Anesthesiology |
| Schwarz | Lilian | MD, PhD | University Hospital of Rouen, France | Digestive Surgical Oncology |
| Grognu | Alexandre | MD | University Hospital of Rouen, France | Anesthesiology |
| Orry | David | MD | Georges François Leclerc Cancer Center, Dijon, France | Surgical Oncology |
| Cogne | Kevin | MD | Georges François Leclerc Cancer Center, Dijon, France | Anesthesiology |
| Ayav | Ahmet | MD, PhD | University Hospital of Nancy, France | Digestive Surgical Oncology |
| Guerci | Philippe | MD, PhD | University Hospital of Nancy, France | Anesthesiology |
| Muscari | Fabrice | MD, PhD | Rangueil University Hospital, Toulouse, France | Digestive Surgical Oncology |
| Porta Bonete | Guillaume | MD | Rangueil University Hospital, Toulouse, France | Anesthesiology |
| Mauvais | François | MD | Simone Veil Hospital, Beauvais, France | Digestive Surgery |
| Hamadouche | Faiza | MD | Simone Veil Hospital, Beauvais, France | Anesthesiology |
| Passot | Guillaume | MD, PhD | Pierre Bénite University Hospital, Lyon, France | Digestive Surgical Oncology |
| Baffeleuf | Bruno | MD | Pierre Bénite University Hospital, Lyon, France | Anesthesiology |
| Trelles | Nelson | MD | René-Dubos Hospital, Cergy-Pontoise, France | Digestive Surgery |
| Benamara | Baya | MD | René-Dubos Hospital, Cergy-Pontoise, France | Anesthesiology |
| Venara | Aurelien | MD, PhD | University Hospital of Angers, France | Digestive Surgical Oncology |
| Lasocki | Sigismond | MD, PhD | University Hospital of Angers, France | Anesthesiology |
| Benoist | Stéphane | MD, PhD | Bicêtre University Hospital, Le Kremlin-Bicêtre, France | Digestive Surgical Oncology |
| De Montblanc | Jacques | MD | Bicêtre University Hospital, Le Kremlin-Bicêtre, France | Anesthesiology |
| Messager | Mathieu | MD | Gustave Dron Hospital, Tourcoing, France | Digestive Surgery |
| Alluin | Laurence | MD | Gustave Dron Hospital, Tourcoing, France | Anesthesiology |
| Fuks | David | MD, PhD | Cochin University Hospital, Paris, France | Digestive Surgical Oncology |
| Dallel | Mohamed Sabri | MD | Cochin University Hospital, Paris, France | Anesthesiology |
| Borraccino | Baptiste | MD | Hospital of Auxerre, France | Digestive Surgery |
| Patrigeon | René Gilles | MD | Hospital of Auxerre, France | Anesthesiology |
| Trésallet | Christophe | MD, PhD | Avicenne University Hospital, Paris, France | Digestive Surgical Oncology |
| Zogheib | Elie | MD, PhD | Avicenne University Hospital, Paris, France | Anesthesiology |
| Valverde | Alain | MD | La Croix Saint Simon Hospital, Paris, France | Digestive Surgery |
| Devys | Jean-Michel | MD | La Croix Saint Simon Hospital, Paris, France | Anesthesiology |
| Souche | François-Régis | MD, PhD | University Hospital of Montpellier, France | Digestive Surgical Oncology |
| De Jong | Audrey | MD, PhD | University Hospital of Montpellier, France | Anesthesiology |
| Herrero | Astrid | MD. PhD | University Hospital of Montpellier, France | Digestive Surgical Oncology and Liver Transplantation |
| Gaujoux | Sébastien | MD, PhD | Pitié Salpêtrière University Hospital, Paris, France | Digestive Surgical Oncology |
| Eyraud | Daniel | MD, PhD | Pitié Salpêtrière University Hospital, Paris, France | Anesthesiology |
| Lefevre | Jérémie | MD, PhD | Saint-Antoine University Hospital, Paris, France | Digestive Surgical Oncology |
| Pardo | Emmanuel | MD | Saint-Antoine University Hospital, Paris, France | Anesthesiology |
